# Improving Outpatient Cancer Care in Gynecologic Oncology: Understanding Patient Preferences During Their Waiting Room Experience

**DOI:** 10.3390/curroncol33080447

**Published:** 2026-07-26

**Authors:** Latteefah Alnaeem, Wafa A. Almohri, Clare Reade, Waldo Jimenez, Sarah Mah, Andra Nica, Julie Nguyen, Lua R. Eiriksson

**Affiliations:** 1 Gynecology Oncology, McMaster University, Hamilton, ON L8S 4L8, Canada; 2 Obstetrics and Gynecology, King Faisal University, Alahsa 31982, Saudi Arabia; 3 Mathematics Department, Taibah University, Al-Madinah Al-Munawarah, Saudi Arabia

**Keywords:** gynecologic oncology, waiting room, patient satisfaction, continuity of care

## Abstract

Background: Waiting-room time is an important and neglected element of the outpatient experience in the field of oncology, which carries far-reaching consequences for overall patient satisfaction, perceptions of quality, and healthcare experience. Methods: We carried out a cross-sectional, mixed-methods study, conducted at the Juravinski Cancer Centre in Hamilton, Canada, from October to December 2024. Results: We studied 418 patients with a mean age of 61.6 years. Overall, 59.6% of respondents had spent less than 30 min in the waiting room, while 56.2% accepted a delay of 15–30 min. Waiting for more than 30–45 min was perceived as long by 61% of participants. In regards to physician choice, 59.8% of respondents preferred to be seen by their known provider, while 30.9% were ready to meet another one, particularly in terms of scheduled regular follow-up and urgent appointments followed by consultations. Interestingly, chemotherapy visits were the least common type of appointment where patients agreed to see different providers. A significant association was identified between appointment types and readiness to change physicians (*p* < 0.05). Although 78.7% agreed that delays were caused by complex conditions, only 29.2% received sufficient information about delays. Conclusions: Most gynecologic oncology patients want continuity of care, yet many are flexible with consultations, follow-up, and urgent visits. In outpatient clinics, patient-centered scheduling, balancing efficiency and patient satisfaction with better communication is an objective for system improvements.

## Simple Summary

This study analyzes patients’ perspectives on waiting times in gynecologic oncology clinics at the Juravinski Cancer Centre, with a particular emphasis on the influence on continuity of care and general satisfaction. Many participants reported waiting for less than 30 min (59.6%). Overall, 56.2% of participants agreed that the maximum limit for a satisfactory waiting period is 15–30 min. Most participants believed that waiting for more than 30–45 min is too long (61%). Many patients would rather wait for their regular provider; however, 30.9% were able to see another doctor during routine appointments. Despite the fact that 78.7% of the participants linked waiting times to the level of case difficulty, 29.2% felt they did not have enough information about the reason for delays.

## 1. Introduction

Time spent in the waiting room is a common and often unavoidable component of outpatient healthcare. In specialty clinics, patients typically arrive on time for scheduled appointments yet may experience delays before being seen by their provider. A substantial body of literature demonstrates that the amount of time patients spend in the waiting room plays a central role in shaping patient satisfaction, perceptions of care quality, and trust in healthcare services [1,2,3,4,5,6,7]. Importantly, multiple studies emphasize that waiting-room time is not merely an operational inconvenience but a meaningful part of the patient experience that warrants focused examination.

Prior research across diverse outpatient settings has consistently identified waiting-room time as a key driver of patient satisfaction. In specialty clinics and surgical settings, longer waiting-room times are associated with lower satisfaction scores, even when clinical care is rated highly [5,6,7]. McMullen and Netland demonstrated that waiting-room time was one of the strongest predictors of overall satisfaction in an ophthalmology clinic [7], while Leddy et al. found that patients’ willingness to wait was limited and strongly influenced their perception of care [6]. Similar findings have been reported in musculoskeletal, endocrine, and surgical outpatient populations [8,9,10,11].

Beyond duration alone, studies have highlighted the psychological dimensions of waiting. Bull et al. described waiting as an experiential process shaped by uncertainty, lack of information, and perceived fairness, rather than minutes alone [2].

Sheridan et al. referred to waiting-room time as the “true penalty” of outpatient care, demonstrating that longer waits had a measurable negative impact on patient satisfaction even in high-performing clinics [3].

Educational or informational interventions delivered during the waiting period have been shown to partially mitigate dissatisfaction, reinforcing the idea that the waiting room itself is a critical site for intervention [1].

In oncology care, outpatient visits are frequent, emotionally charged, and often prolonged. Patients may attend clinics repeatedly for consultations, treatment planning, chemotherapy, surveillance, and follow-up. At each visit, patients typically wait in the waiting room before being seen, making waiting-room time a recurring feature of the cancer care journey. Thomas et al. (1997) identified waiting as the single worst aspect of the oncology outpatient experience for more than one-quarter of patients, underscoring the salience of this issue within cancer care settings [9].

Despite this, waiting-room time in oncology clinics remains underexamined relative to other aspects of care delivery [12,13].

## 2. Rationale

Patients with gynecologic malignancies represent a distinct and potentially vulnerable subgroup of the oncology population. Their care often involves invasive diagnostic procedures, sensitive physical examinations, complex treatment discussions, and long-term surveillance. These encounters frequently exceed scheduled appointment times, leading to cumulative delays for subsequent patients [14]. Overbooking practices to accommodate high patient volumes and the involvement of trainees may further contribute to prolonged waiting-room times [15,16].

While prior studies clearly establish that waiting-room time negatively affects patient satisfaction, most research has focused on general outpatient populations or non-oncologic specialties [3,4,5,6,7,8]. There is limited evidence examining how gynecologic oncology patients perceive waiting-room time specifically, how they define a “prolonged” wait, and how waiting-room delays influence their overall clinic experience. Moreover, it remains unclear whether patients would be willing to trade continuity of care for shorter waiting-room times and whether this willingness varies by appointment type or clinical context [17,18].

Prolonged waiting-room time may also impose tangible burdens on patients beyond dissatisfaction. Extended waits can result in increased financial costs such as parking fees, physical discomfort, fatigue, pain, and disruption to work or caregiving responsibilities. These burdens may be amplified in oncology patients who require frequent visits and who may already be experiencing physical or emotional distress. Despite recognition of these issues in the broader outpatient literature [2,5,8], their impact has not been adequately explored within gynecologic oncology clinics.

This study was undertaken to address these gaps by focusing specifically on waiting-room time as the primary area of interest. By examining patient perceptions of waiting-room duration, contributors to delays, communication during waits, and preferences regarding continuity of care versus shorter waiting times, this research aims to clarify how waiting-room time impacts the experience of gynecologic oncology outpatients [19,20].

## 3. Significance of the Study

This study contributes to an important yet underexplored area of oncology care by centering the waiting-room experience of gynecologic oncology outpatients. The existing literature demonstrates that waiting-room time is a modifiable factor that strongly influences patient satisfaction across healthcare settings [1,2,3,4,5,6,7]. However, oncology-specific data, particularly for gynecologic oncology patients, remain limited.

By clearly defining the problem as the amount of time patients spend in the waiting room, this study provides actionable insights for outpatient oncology services. Understanding what patients consider a prolonged waiting-room time, how delays affect their experience, and under what circumstances they are willing to see alternate providers can inform clinic scheduling, communication strategies, and models of care that balance efficiency with patient-centeredness [21,22,23].

Furthermore, positioning waiting-room time within the broader context of patient time burden highlights opportunities to reduce unnecessary strain on patients receiving complex cancer care. The findings from this study may guide quality improvement initiatives aimed at improving patient experience without compromising clinical outcomes [3,24]. The structured approach and focus on waiting-room time also allow this study framework to be adapted for subsequent projects in other oncology outpatient settings.

## 4. Methodology

### 4.1. Research Design

This research study used a mixed-methods cross-sectional design. The quantitative part provides descriptive and inferential statistics that contribute to assigning meaning to the mathematical relevance of the correlation between variables [25]. Qualitative elements will provide additional and more in-depth information and theoretical explanations that will add a higher level of meaning to the statistics than would be obtained based on quantitative data. The plurality of research designs is crucial for developing a more thorough understanding of the variables and formulating the arguments.

### 4.2. Study Setting and Population

This study was conducted at the Juravinski Cancer Centre, Hamilton Health Sciences, located in Hamilton, Canada. Data collection occurred over a duration of 12 weeks, from 7 October to 29 December 2024. Preparation commenced on 25 September 2024, and the close-out was finalized on 30 December 2024. The inclusion criteria included patients attending outpatient gynecologic oncology clinics at the Juravinski Cancer Centre who were 18 years and older, could read and comprehend English, and were willing to participate in the study.

### 4.3. Sampling Techniques

#### 4.3.1. Sample Size

Sample size determination stemmed from the operational data. The gynecologic oncology clinic is open five days a week, and each clinic accommodates approximately 42 patients. This includes four new patient consultations, ten chemotherapy patients, three post-operative patients, sixteen patients on surveillance, four urgent appointments, and five patients under review for investigations and treatment planning. It was estimated that the weekly clinic volume would be 252 patients, and that the total number of patients seen during the 12-week study period would be 3024. The required sample size was calculated to be approximately 341 patients based on Cochran’s method [26] when handling small populations, a 95 percent confidence interval, and a 5 percent error. The final sample of 418 surveys was larger than the recommended minimum sample and increased the statistical power of the study.

#### 4.3.2. Sampling Technique

The research used a convenience sampling technique, meaning that the patients were selected according to their availability during clinic visits and their enthusiasm to participate.

#### 4.3.3. Instrument Development

The student principal investigator (LA) and the supervising principal investigator (LRE) worked together to create a structured, paper-based questionnaire. The questionnaire contained 16 closed-ended questions and one open-ended question for a total of 17 questions.

### 4.4. Data Collection Procedures

#### 4.4.1. Data Collection Process

Unit clerks at the registration desk gave out paper-based surveys to eligible patients. Patients were informed about the study’s voluntary nature and their opportunity to withdraw prior to submission. Individuals deposited their completed questionnaires in a locked box, which was emptied on a daily basis. Thereafter, to enhance efficiency, replies were instantly input into an Excel spreadsheet and securely saved on an encrypted USB stick with a password. The paper surveys have been safely retained in author custody.

#### 4.4.2. Data Analysis

Before being analyzed, the obtained data underwent a series of methodical cleaning steps, including cross-checking the data for missing or incomplete answers.

This step was crucial for ensuring data completeness, which included determining the range and consistency of numbers, coding open-ended answers for theme analysis, and creating new variables for further analysis. We derived additional analyzable fields from existing responses to support summary statistics and comparisons (e.g., collapsing categories, creating indicators like “long wait,” or creating composite scores from multiple items). After inputting the data, we checked the spreadsheet to ensure all replies were within the allowable answer possibilities and that “I don’t know” and “prefer not to answer” responses were treated uniformly. Then, we randomly picked 100 of the 418 surveys (24%) and compared each paper survey question-by-question to the Excel input. To ensure data accuracy, discrepancies were addressed, and revised entries were reviewed again. To prevent repeat surveys from the same participant, each time patients checked in/out, the clerk offered questionnaires and requested that patients only complete one survey during the study. We limited distribution to clinic sessions and analyzed daily survey results to prevent duplicate distribution and validate the correctness of select surveys. The study utilized IBM SPSS Statistics (version 26) to analyze data. The analysis utilized chi-square tests of independence to examine the links between categorical variables, especially the link between appointment type and willingness to see an alternate gynecologic oncologist. Assumptions for testing were examined and determined based on established criteria [27]. Criteria included expected frequencies larger than five, whether there were independent observations, and an adequate sample size for analyzing the contingency table.

This study used thematic coding for the open-ended responses based on Braun & Clarke’s six-phase process [28]. The first step involved reviewing the questions and identifying the responses through text coding. For the third stage, the established themes were grouped based on their ordinary meaning and relation to the study objectives, reviewed for accuracy, and then defined. The last phase was report writing. A selection of answers were coded to determine inter-rater reliability, with any discrepancies addressed via discussion and consensus. The ultimate theme framework was created through a process of coding, evaluating, and revising that was repeated until thematic saturation was reached.

## 5. Ethical Considerations

This study was approved by the Hamilton Integrated Research Ethics Board (HIREB) no. 17383 issued on 9 September 2024. The patient information and consent form informed the survey participants that their involvement was voluntary, that they could withdraw from the study prior to submitting their responses, and that questionnaire completion and submission implied that they agreed to take part in the study.

## 6. Results

There were 418 gynecologic oncology patients who completed the survey during the study period. Patient age ranged from 24 to 93 years, with an average age of 62 +/− 13 years. The patient’s spouse or partner was the most common companion (35.6%), closely followed by those who come alone (32.3%). The majority of responders (58.1%) had completed college or university. The most prevalent diagnoses in patients who completed the survey were ovarian/fallopian tube cancers (33%) and uterine cancer (29.4%), which accounted for 62.4% of all cases (Table 1).

Among the participants, the most prevalent intervals between visits included 3–6 months (34%) and 3–4 weeks (33.3%). It should be noted that fewer participants were visiting for the first time (12.4%) or visited once a week (3.3%) (Table 2).

Conversely, the results in Table 3 and Table 4 indicate that fewer than half of the patients (48.6%) expressed significant comfort with being examined by a gynecologic oncology trainee (e.g., fellow, resident) for educational purposes. Most patients (72.2%) believed that being seen by a trainee would not make any difference in their waiting time.

For the preferred waiting location prior to meeting the healthcare provider, it is evident that the majority preferred waiting in the common waiting area alongside other patients (41.6%), followed by waiting in the examination room (33.6%). A minority of the patients (14.1%) preferred to wait in any part of the hospital until summoned (Table 5).

The results in Table 6 indicate a high level of overall satisfaction with the waiting area. Almost half of the respondents (48.8%) reported feeling moderately comfortable, and 43.8% reported feeling very comfortable. A tiny minority found the waiting room uncomfortable (3.3%) or very uncomfortable (1.2%).

Table 7 presents patients’ evaluations of different waiting area facilities. Overall, cleanliness was the most positively evaluated by 72% of respondents, as either excellent (29%) or good (43%). Seat comfort was also perceived as a plus, with 58% of respondents giving excellent (15%) or good (43%) scores. Also, 51% rated Wi-Fi access as good, while 30% did not answer. Reading materials, drinks, and entertainment opportunities were rated lower; reading materials were regarded as poor or very poor by 27% of the respondents, and refreshments and entertainment were rated negatively by 23% and 23% of the participants, respectively. A large majority of respondents also did not rate several amenities, including Wi-Fi (30%), refreshments (29%), and entertainment services (28%).

The following Table 8 shows the patients’ willingness to see another gynecologic oncologist in order to decrease waiting periods. Most participants (59.8%, *n* = 250) would prefer to continue visiting their existing healthcare provider even with potential delays. In contrast, 13.9% (*n* = 58) said they would be prepared to see a different physician if it meant shorter wait times, while 17.0% (*n* = 71) said their decision would depend on the type of appointment. Another 9.3% (*n* = 39) were neutral towards seeing either physician. When participants were asked about types of appointments that might prompt them to consider seeing a different physician, routine follow-up visits after treatment were most frequently identified (30.0%, *n* = 160). This was followed by follow-up appointments for test results (22.1%, *n* = 118), urgent appointments (20.0%, *n* = 107), consultations (16.1%, *n* = 86), and chemotherapy visits (11.8%, *n* = 63).

Table 9 presents the distribution of appointment types among individuals who reported that their willingness to see another gynecologic oncologist was dependent on appointment type. Of the respondents who selected the appointment-dependent option (*n* = 67), the most common type of appointment was follow-up visits after treatment (59.7%), followed by follow-up visits for test results (46.3%) and urgent appointments (38.8%). Additionally, 31.3% of the replies were chemotherapy visits, while consultations had the lowest proportion at 23.9%. For respondents who did not specify that their decision was based on appointment type (*n* = 222), the most common appointment type was follow-up visits after treatment (54.1%), followed by follow-up visits for test results (39.2%), urgent appointments (36.5%), consultations (31.5%), and chemotherapy visits (18.9%). Overall, follow-up appointments post-treatment were the most common type of appointment reported by participants (55%), while chemotherapy visits were the least common (22%).

**Figure 1:**
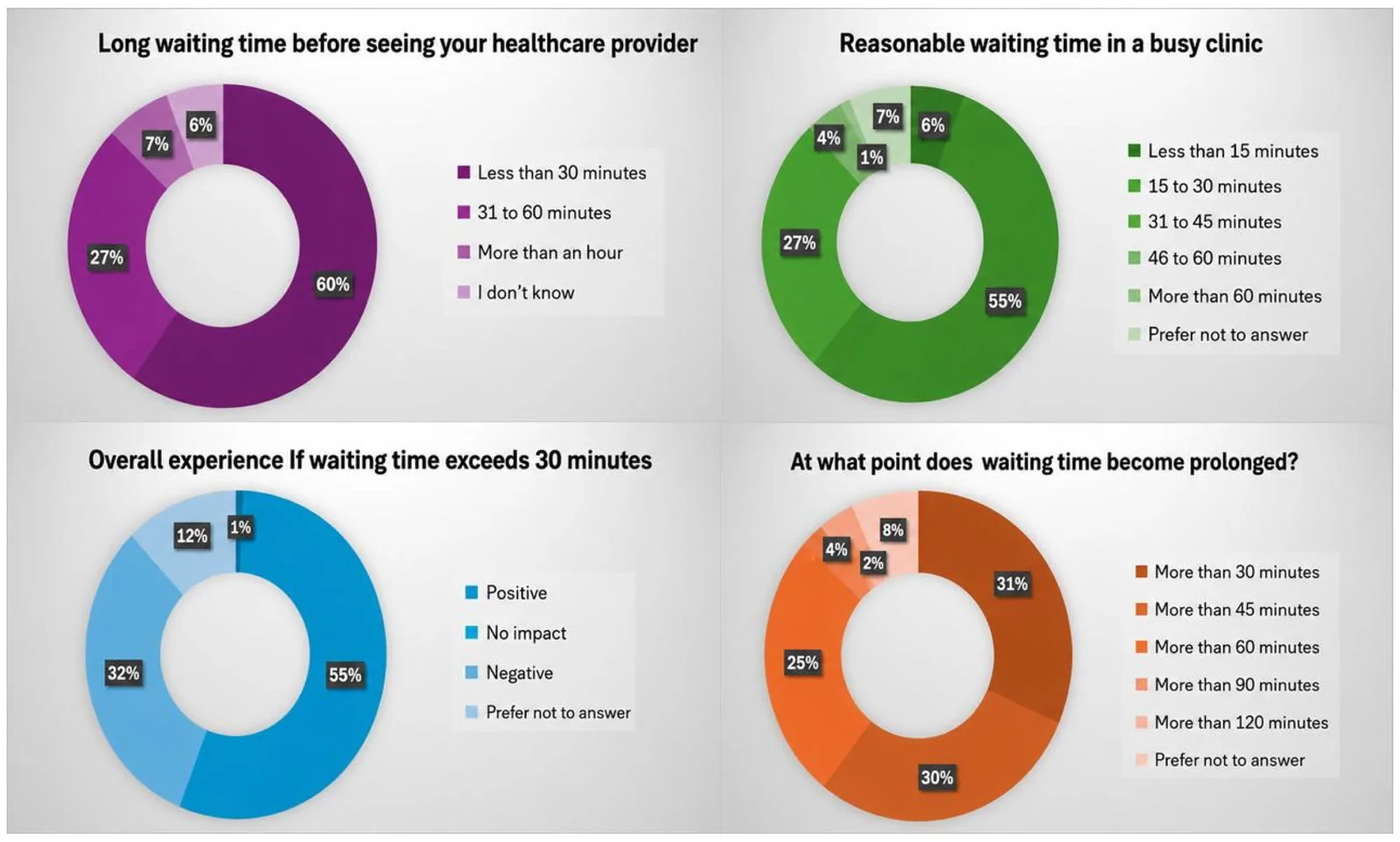
Patient perceptions of prolonged waiting-room time.

Most gynecologic oncology patients reported that their usual waiting-room time is less than 30 min (59.6%), whereas 27.3% wait between 31 to 60 min (Figure 1). The majority of patients (56.2%) believe that from 15 to 30 min is a reasonable waiting-room time in a busy oncology clinic. More than half of patients (54.8%) reported that there was no impact from increased waiting-room times, while (31.6%) of patients reported a negative experience. Waiting beyond 30 to 45 min was considered a prolonged waiting-room time for 61% of patients; by 60 min, 86% of patients agree that waiting-room time has become prolonged.

Most of the participants (60.4%) preferred free parking for the day as the most desired compensation for extended waiting times. Complimentary refreshments (19.8%) and discount vouchers (9.5%) were less popular, while apologies from staff and lottery entries were seldom favored (Table 10).

The theme most frequently identified from patient suggestions was communication and information (25.8%), reflecting patients’ desire for more information about delays and estimated waiting periods. The next most popular theme was service appreciation (20.2%), which suggests that although there were worries about waiting time, many patients still felt content with the care they received. Other themes were scheduling and time management (18.0%), environmental improvements (16.9%), and practical support measures (13.5%). The patients’ suggestions were mainly about better communication, improved scheduling techniques, and greater comfort during waiting times (Table 11).

## 7. Discussion

This research examined waiting times and continuity of service among 418 gynecologic oncology outpatients at Juravinski Cancer Centre. The mean age of the health seekers was 61 years, and they were assisted and supported by relatives. Overall, 52.6% of the enrolled patients were receiving routine follow-up. This article presents valuable information regarding patient attitudes toward in-clinic waiting times and preferences for continuity of care in gynecologic oncology. Patients believed that 15–30 min waiting times are fair but 30–45 min waiting times are too long, setting objective performance demands for the clinic. Around 60% of patients waited less than 30 min; a third of patients said they became frustrated with longer wait times. This means that waiting times, both real and perceived, need to be shorter.

It is notable that most of the study participants, almost 60%, prefer to see their regular healthcare provider on each visit. The statistically significant difference between appointment type and the desire to see other providers shows well-developed patient logic when it comes to choosing when continuity is most important. Routine follow-ups have the highest flexibility (59.7%) for those who are willing to see a different provider, while interestingly, consultations (30%) and chemotherapy visits (22%) show less flexibility, as patients do view the oncologist’s reputation and relational continuity as critical.

Most patients (78.7%) believed that the complexity of the situation, not incompetence, caused delays. However, only 29.2% of patients believed they received communication about delays, while 25.8% requested more frequent updates. This shows that being open and honest, whether via front-desk workers or digital technologies, might help build trust. Given the financial difficulties associated with many cancer consultations, 60.4% of patients favored free parking as a reward, indicating that resources should focus on practical patient support rather than luxuries. Furthermore, most participants preferred hygiene to entertainment, indicating that luxury was not relevant to cancer patients and rather, basic comfort gave them relief. Therefore, facilities should focus on practical help more than symbolic gestures.

The results replicate earlier research findings. Patient satisfaction is more accurately assessed using perceived waiting time rather than real waiting time [7]. Communication and continuity of care are still critical [10,11], and research shows that regular updates may help keep people satisfied even when they have to wait a long period [16]. To our knowledge, our study is the first prospective study to show patients’ preference for continuity of care from their regular provider in the field of gynecologic oncology. While accepting flexibility for normal follow-up, patients are more restricted for chemotherapy visits, reflecting the emotional support and link patients experience through continuity of care [21,29]. Research on servicescapes also backs up the findings, showing that cleanliness and sitting are associated with satisfaction.

## 8. Study Limitations

The monocentric design of this study limits translatability to different centers with operationally distinct and variably characteristic cancer centers and patient populations. Despite the constraints of convenience sampling regarding representativeness, the considerable sample size (*n* = 418) alleviates the effects of sampling bias and improves the external validity of the findings for this study population. The response rate introduces issues of representativeness; yet the substantial absolute sample size is likely to ensure sufficient statistical power for primary analyses. Patients who have had a good or bad experience can be more motivated to answer questionnaires, which leads to response bias. The cross-sectional design collects impressions at a single point and does not allow for seasonal changes or changes in clinic practices [30]. Despite the differences between subjective and objectively measured waiting times, research indicates a stronger correlation between subjective impressions and patient satisfaction. Lastly, participants were recruited on a voluntary basis which may have caused selection bias. In addition, the uneven distribution of participants throughout treatment stages prevented subgroup analysis and might have influenced the generalizability of the findings.

## 9. Future Research

These findings present many research opportunities, such as conducting multicenter studies in various cancer environments. Another benefit would be the ability to assess the generalizability of trends to different oncologic specialties and patients. Secondly, an important factor when making judgments about competing patient and provider demands is considering patient visit patterns. Furthermore, a cost–benefit analysis, possibly within the context of proposed treatments, could be advantageous in defining the rate at which patient wait times could be decreased.

## 10. Conclusions

The targeted population in this study were oncology patients undergoing treatment in a cancer center during outpatient visits. Our results suggest that most gynecologic oncology patients would prefer continuity of care and better communication of waiting times, yet many are flexible with routine follow-up and urgent visits. This information can be used to balance efficiency with patient preferences.

## Figures and Tables

**Table 1:** Participants’ demographics data.

		No. (*n* = 418)	
Age (in years)	Min–Max	24–93	
	Mean ± SD	61.56 ± 13.01	
		Frequency	%
Who accompanied patient to clinic appointment	I attend alone.	135	32.3%
	Spouse or partner.	149	35.6%
	Parent.	17	4.1%
	Other family member.	88	21.1%
	Friend.	19	4.5%
	Healthcare aide or assistant.	3	0.7%
	No one accompanies me regularly, but it varies.	7	1.7%
Education level	Primary/High School	165	39.5%
	College/University graduate	243	58.1%
	Advance study (PHD)	10	2.4%
Cancer diagnosis	Ovarian and/or fallopian cancer	138	33.0%
	Uterine cancer	123	29.4%
	Cervical cancer	66	15.8%
	Vaginal cancer	13	3.1%
	Vulvar cancer	29	6.9%
	Not yet diagnosed with cancer	49	11.7%

**Table 2:** Visit frequency.

Question	Choices	Frequency	%
Frequency of outpatient oncology clinic visit	My first visit	52	12.4
	Weekly	14	3.3
	Every three to four weeks	139	33.3
	Every three to six months	142	34
	I am not sure for now	71	17

**Table 3:** Comfort with different-rank providers.

Question	Choices	Frequency	%
Comfort with being seen by a different-rank healthcare provider for teaching purposes.	Very comfortable—I am fine with being part of the teaching process.	203	48.6
	Somewhat comfortable—I prefer to see my primary provider but understand the need to educate learners.	127	30.4
	Neutral	32	7.7
	Not comfortable at all—I would refuse to see a learner if given the option.	16	3.8
	Prefer not to answer.	40	9.6

**Table 4:** Perceived impact of being seen by a fellow or resident on waiting-time duration.

Question	Choices	Frequency	%
Perceived effect of treatment by a fellow or a resident for teaching purposes on longer waiting time	Yes, significantly longer.	43	10.3
	No difference.	302	72.2
	No, it makes it shorter.	26	6.2
	Prefer not to answer.	47	11.2

**Table 5:** Preference for waiting time.

Question	Choices	Frequency	%
Preference for waiting prior to being seen by healthcare provider	In a shared waiting room with other patients.	183	41.60%
	In an examination room.	148	33.60%
	From any place in the hospital until called in for my appointment (for example, with a pager).	62	14.10%
	I do not know.	47	10.70%

**Table 6:** Comfort with the waiting area in the oncology clinic.

Question	Choices	Frequency	%
Comfort with the waiting area in the oncology clinic	Very comfortable	183	43.8
	Moderately comfortable	204	48.8
	Uncomfortable	14	3.3
	Very uncomfortable	5	1.2
	I do not know	12	2.9

**Table 7:** Patient ratings of waiting area amenities.

	Excellent	Good	Average	Poor	Very Poor	Prefer Not to Answer
**Seat comfort**	15%	43%	30%	2%	0%	10%
**Cleanliness**	29%	43%	16%	1%	0%	12%
**Reading materials**	3%	17%	25%	17%	10%	18%
**Wi-Fi**	17%	34%	17%	2%	2%	30%
**Refreshments**	8%	20%	20%	12%	11%	29%
**Entertainment (TV, music)**	3%	17%	29%	14%	9%	28%

**Table 8:** Willingness to see an alternative gynecologic oncologist to shorten wait times.

**Willing to see another physician to reduce wait time**	Yes, I prefer shorter wait times	58	13.9%
	No, I prefer to see my regular healthcare provider	250	59.8%
	It is appointment dependent	71	17.0%
	I am indifferent	39	9.3%
**Willingness to see a different physician by appointment type**	Consultation	86	16.1%
	Routine follow-up after treatment	160	30.0%
	Urgent appointment (requested by the patient)	107	20.0%
	Follow-up for test results	118	22.1%
	Chemotherapy visit	63	11.8%

**Table 9:** Results for patients who responded “It would depend on the type of appointment” for willing to see another physician vs. appointment types.

Appointment Type	Depends on the Type of Appointment		Total
	Yes	No	
Consultation	16 (23.9%)	70 (31.5%)	86 (30%)
Follow-up after treatment	40 (59.7%)	120 (54.1%)	160 (55%)
Urgent appointment	26 (38.8%)	81 (36.5%)	107 (37%)
Follow-up for test results	31 (46.3%)	87 (39.2%)	118 (41%)
Chemotherapy visit	21 (31.3%)	42 (18.9%)	63 (22%)
Total	67 patients * (100%)	222 patients * (100%)	289 (100%)

* Participants were allowed to select more than one appointment type; therefore, percentages may exceed 100% within categories.

**Table 10:** Compensation preferences.

Compensation Type	Frequency (*n*)	Percentage (%)
Free parking for the day	165	60.4
Complimentary refreshments	54	19.8
Discount vouchers	26	9.5
Apology from clinic staff	20	7.3
Raffle entry for gift basket	8	2.9

**Table 11:** Thematic analysis of patient suggestions.

Theme	Frequency (*n*)	Percentage (%)	Description
Communication and Information	23	25.8	Requests for better communication about delays, estimated wait times, and regular updates during waiting periods
Service Appreciation	18	20.2	Expressions of gratitude for current care quality and recognition of staff efforts
Scheduling and Time Management	16	18.0	Suggestions for more realistic appointment scheduling, reducing overbooking, and better time allocation per patient
Environmental Improvements	15	16.9	Requests for physical space enhancements including functional televisions, improved privacy, better lighting, and cleaner facilities
Practical Support	12	13.5	Suggestions for tangible support such as refreshments, paging systems to allow mobility, and parking assistance
No Suggestions/Satisfaction	5	5.6	Patients expressing satisfaction with current services and having no suggestions for improvement

## Data Availability

The original contributions presented in this study are included in the article. Further inquiries can be directed to the corresponding author(s).
